# Nephron filtration rate and proximal tubular fluid reabsorption in the Akita mouse model of type I diabetes mellitus

**DOI:** 10.12688/f1000research.2-83.v1

**Published:** 2013-03-11

**Authors:** Jurgen Schnermann, Mona Oppermann, Yuning Huang

**Affiliations:** 1 National Institute of Diabetes and Digestive and Kidney Diseases, NIH, Bethesda, MD, 20892, USA; 2 Children's Hospital, University Medical Center, University of Regensburg, Regensburg, Germany

## Abstract

An increase of glomerular filtration rate (hyperfiltration) is an early functional change associated with type I or type II diabetes mellitus in patients and animal models. The causes underlying glomerular hyperfiltration are not entirely clear. There is evidence from studies in the streptozotocin model of diabetes in rats that an increase of proximal tubular reabsorption results in the withdrawal of a vasoconstrictor input exerted by the tubuloglomerular feedback (TGF) mechanism. In the present study, we have used micropuncture to assess single nephron function in wild type (WT) mice and in two strains of type I diabetic Ins2+/- mice in either a C57Bl/6 (Akita) or an A1AR-/- background (Akita/A1AR-/-) in which TGF is non-functional. Kidney glomerular filtration rate (GFR) of anesthetized mice was increased by 25% in Akita mice and by 52% in Akita/A1AR-/-, but did not differ between genotypes when corrected for kidney weight. Single nephron GFR (SNGFR) measured by end-proximal fluid collections averaged 11.8 ± 1 nl/min (n=17), 13.05 ± 1.1 nl/min (n=23; p=0.27), and 15.4 ± 0.84 nl/min (n=26; p=0.009 compared to WT; p=0.09 compared to Akita) in WT, Akita, and Akita/A1AR-/- mice respectively. Proximal tubular fluid reabsorption was not different between WT and diabetic mice and correlated with SNGFR in all genotypes. We conclude that glomerular hyperfiltration is a primary event in the Akita model of type I diabetes, perhaps driven by an increased filtering surface area, and that it is ameliorated by TGF to the extent that this regulatory system is functional.

## Introduction

The development of both type I and type II diabetes mellitus (DM) is often associated with an increase of glomerular filtration rate (GFR) (usually referred to as glomerular hyperfiltration), and the same phenomenon has been observed in various experimental models of DM ^1, 2^. The issue of diabetic hyperfiltration has attracted substantial interest because of the evidence that the occurrence of hyperfiltration may have some value in predicting the development of diabetic nephropathy ^3^. This concept seemed plausible because of the evidence that hyperfiltration may be caused by increased glomerular capillary pressure and that intraglomerular hypertension represents a general risk factor for glomerular disease ^4^. Despite the continuing debate about the reality behind the link between diabetic hyperfiltration and diabetic nephropathy, the issue of the causation of glomerular hyperfiltration has been intensely pursued in rodent models of diabetes. Among the proposed mechanisms responsible for diabetic hyperfiltration, relaxation of afferent arterioles in response to reduced input from tubuloglomerular feedback (TGF) has played a prominent role.

TGF is an intrarenal regulatory system that operates at the level of the juxtaglomerular apparatus, and that translates changes in NaCl concentration at a distal tubular site, probably the macula densa, into inverse changes of glomerular capillary pressure and nephron filtration rate ^5^. Two different concepts have been advanced as to how TGF may be involved in the dysregulation of GFR in DM. One hypothesis argues that the primary process is the growth of the proximal tubule leading to enhanced water and solute reabsorption with the consequence that NaCl delivery to the macula densa decreases, the TGF-imposed vasomotor tone relaxes, and glomerular capillary pressure and GFR increase ^6^. This “tubulo-centric” concept has been supported by substantial experimental evidence coming for the most part from experiments in rats with streptozotocin-induced type I DM ^6^. Alternatively, it has been suggested that diabetic hyperfiltration is primary, driven by structural changes and/or by largely unknown derangements in the spectrum of vasoactive mediators, and that TGF serves as a mechanism that prevents the full extent of the effects of these abnormalities on GFR ^7^. These two concepts are not easily reconcilable because vasorelaxation is caused by a normally functioning TGF in the first, whereas, in the second, vasorelaxation is TGF-independent and is in fact counteracted by it to the extent TGF is functional. This “glomerulo-centric” theory has found support in the finding that type I diabetic mice of the Akita strain without a functional TGF system, achieved by breeding the Ins2 mutation of the Akita mice into the TGF-less A1 adenosine receptor (A1AR) null background ^8^, display exaggerated hyperfiltration compared to Akita mice with a presumably intact TGF ^9^.

In a recent extensive review of the complex issues surrounding renal function in diabetic models, it has been argued that the failure to detect a clear TGF relaxation in the Akita mouse model of diabetes might be due to excessively high plasma glucose levels and the inability of proximal tubules to enhance reabsorption sufficiently ^10^. This may prevent distal NaCl levels from falling, thereby maintaining some TGF activation and preventing hyperfiltration. Even though this argument does not explain the exaggerated hyperfiltration in the Akita mice with the A1AR-/- background in which the absence of TGF makes variations of distal NaCl irrelevant, we have taken this argument as an incentive to directly assess proximal fluid reabsorption by micropuncture in Akita diabetic mice with both native and A1AR-/- backgrounds. While confirming the presence of hyperfiltration in the TGF-less diabetic mice, our data show that there are no measurable reductions in the rates of proximal fractional fluid reabsorption between WT mice and diabetic animals with or without TGF. We therefore maintain the view that, at least in this particular model of type I diabetes, TGF serves as a mechanism protecting against the development of uncontrolled hyperfiltration.

## Methods

### Animals

Male Akita mice heterozygous for the Ins2 mutation (Ins2+/–; C57Bl/6 background) from Jackson Laboratories (Bar Harbor, ME, USA) were crossed with female C57Bl/6 WT mice in the NIH animal facility. To generate Ins2+/–/A1AR-/- double mutants, female A1AR-/- mice (C57Bl/6 background) were crossed with male F2 mice heterozygous for both the Ins2 (Akita) and A1AR mutations. All micropuncture experiments were performed in male animals Successful experiments were performed in 16 animals (WT=5, Akita=5, Akita/A1AR-/-=6). Mice were housed in the NIH animal facility at a room temperature of 22°C and a 12 hour dark/12 hour light cycle. Animal experimentation was approved and carried out in accordance with the NIH Guide for the Care and Use of Laboratory Animals. Genotyping for A1AR was done on tail DNA using PCR as described previously ^8^. Genotyping of Ins2 was done by standard PCR (primers: sense TGCTGATGCCCTGGCC TGCT, antisense TGGTCCCACATATGCACATG) using Ampli *Taq* DNA Polymerase (Applied Biosystems, Foster City, CA, USA). The PCR product was then digested for 2 h with Fnu4HI restriction enzyme (Cat# R01785; New England Biolabs, Ipswich, MA, USA) and separated on agarose/ethidium bromide (3% [w/v]) gel to yield bands of 140 bp for WT, and 280 bp for the Ins2 mutation.

### Animal preparation

For micropuncture experiments, mice were anesthetized with 100 mg/kg thiobutabarbital (inactin) intraperitoneally and 100 mg/kg ketamine subcutaneously. Body temperature was maintained at 37.5°C by placing the animals on an operating table with a servo-controlled heating plate. The trachea was cannulated, and a stream of 100% oxygen was blown towards the tracheal tube throughout the experiment. The left carotid artery was catheterized with hand-drawn polyethylene tubing for continuous measurement of arterial blood pressure and blood withdrawal. A hand-drawn polyethylene catheter connected to an infusion pump was inserted into the right jugular vein for an intravenous maintenance infusion of saline at 400 µl/hr. The bladder was cannulated from a suprapubic midline incision for urine collections. Following a flank incision, the kidney was carefully dissected free of surrounding fat and placed in a lucite holder. The opening of the cup at the hilum was obstructed with fat and the kidney was covered with mineral oil.

### Glomerular and tubular function of single nephrons

To measure rates of proximal fluid reabsorption, an infusion of ^125^I-iothalamate (Glofil-125, Iso-Tex Diagnostics, Friendswood, TX, USA; ~40 µCi/hr) was started 20–25 minutes before micropuncture. Nephron filtration and absorption rates were determined by free-flow micropuncture as previously described ^11^. Following tubular identification by dye injection, proximal collections were done in the last surface segment (collection duration 2.5 min in most cases ) using oil-filled pipettes. Fluid volume was determined from column length in a 0.5 µl Drummond microcap. Samples were transferred into a counting vial and radioactivity was determined in a gamma counter (Riastar, Packard Instrument Company, U.S.A.). Blood samples were collected in heparinized 5 µl microcaps at the beginning and at the end of the experiment. Temporal spacing between the two blood samples was between 38 and 55 minutes. Plasma reference values for each tubular sample were obtained by linear interpolation. ^125^I-iothalamate radioactivity was measured in duplicates using 0.5 µl samples of plasma and urine using Drummond 0.5 µl microcaps for sample transfer.

### Statistics

All reported statistical comparisons were made by one way ANOVA using the Bonferroni post hoc test at p<0.05 as showing significance (GraphPad Prism, GraphPad Software Inc., San Diego U.S.A.).

## Results

### Kidney function

A summary of measurements of GFR and a number of functional variables in WT (n=5), Akita (n=5), and Akita/A1AR-/- mice (n=6) during anesthesia is shown in Table 1. As observed previously in conscious mice, GFR was lower in WT than in diabetic mice reaching significance in the Akita diabetic mice with the A1AR deletion. Because body weights (BW) were not different between genotypes, the same increase was observed when GFR was normalized for 100 g of body weight. Interestingly, however, significant differences disappeared when GFR was expressed per kidney weight (KW), reflecting the fact that kidney weights were significantly higher in both groups of diabetic mice compared to WT. As indicated by the increased KW/BW ratio, the increase of kidney weight occurred without similar changes in body weight. It is safe to assume that glucosuria was the cause of the significantly higher urine flows in both strains of Akita mice as shown previously ^12^. There were no significant differences between WT and diabetic mice in mean arterial blood pressure, body weight, or age. An estimate of the number of nephrons filtering at the level of measured SNGFRs (Kidney GFR/SNGFR) suggests that nephron numbers were not significantly different between the genotypes used in this study.

**Table 1.** Kidney function and general metrics of wild type (WT), Akita, and Akita/A1AR-/- double mutant mice.

Parameter	Wild type (n=5)	Akita (n=5)	Akita/A1AR-/- (n=6)
GFR (µl/min)	312.3 ± 21	390 ± 57	487.2 ± 25 *
GFR (µl/min *100g BW)	1135.7 ± 45	1412 ± 201	1786.2 ± 84 **
GFR (µl/min * g KW)	925.9 ± 64	964 ± 161	980.6 ± 36.9
MAP (mm Hg)	95.5 ± 3.7	91 ± 2.2	87 ± 2.4
UV (µl/min)	1.5 ± 0.3	4.1 ± 0.4 **	4.9 ± 0.6 **
BW (g)	27.4 ± 0.9	27.6 ± 0.7	27.3 ± 1
KW (mg)	338.2 ± 10.5	411 ± 27.4 *	496.2 ± 13 **
KW/BW (mg/g)	12.4 ± 0.4	14.9 ± 0.9 *	18.2 ± 0.5 **
Age (wk)	13.3 ± 1.1	17.5 ± 1.5	11.7 ± 1.4
GFR/SNGFR	28127 ± 1972	29615 ± 2910	32016 ± 2240

GFR, glomerular filtration rate; MAP, mean arterial blood pressure; UV, urine flow rate; BW, body weight; KW, kidney weight; GFR/SNGFR, mean GFR divided by mean single nephron GFR (number of functional nephrons for both kidneys).

*p<0.05, **p<0.01 (ANOVA with Bonferroni post hoc test; statistics given for comparison with wild type).

### Nephron function

**Figure 1.**
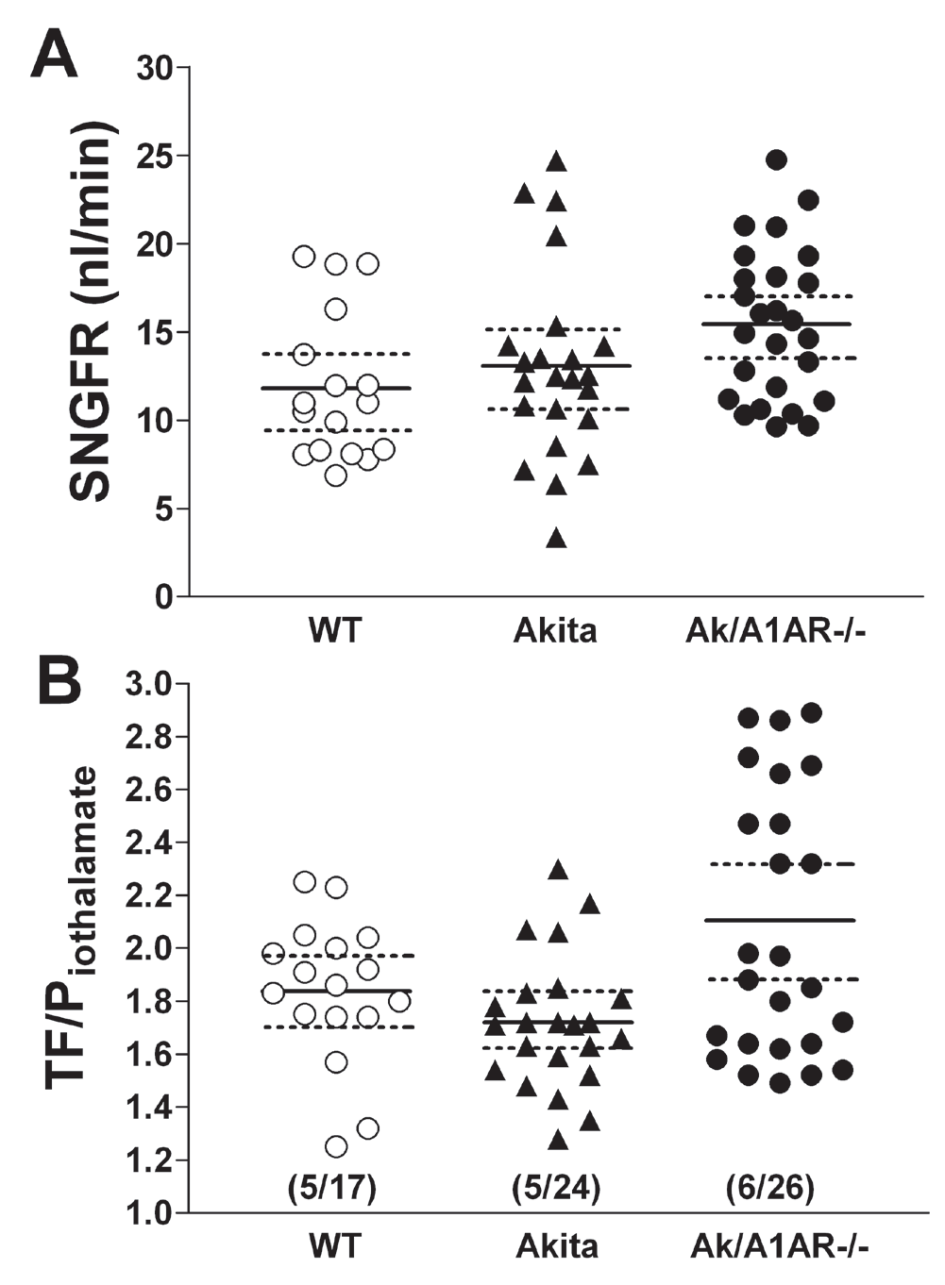
Measurements of single nephron filtration rate (SNGFR) and TF/Piothalamate ratios in wild type (WT), Akita, and Akita/A1AR-/- double mutant mice (Ak/A1AR-/-). **A**: SNGFR in individual tubules; solid lines indicate mean values and broken lines are 95% confidence intervals. **B**: TF/Piothalamate ratios in individual tubules; solid lines are mean values and broken lines are 95% confidence intervals; numbers in brackets are numbers of mice/numbers of tubules.

**Figure 2.**
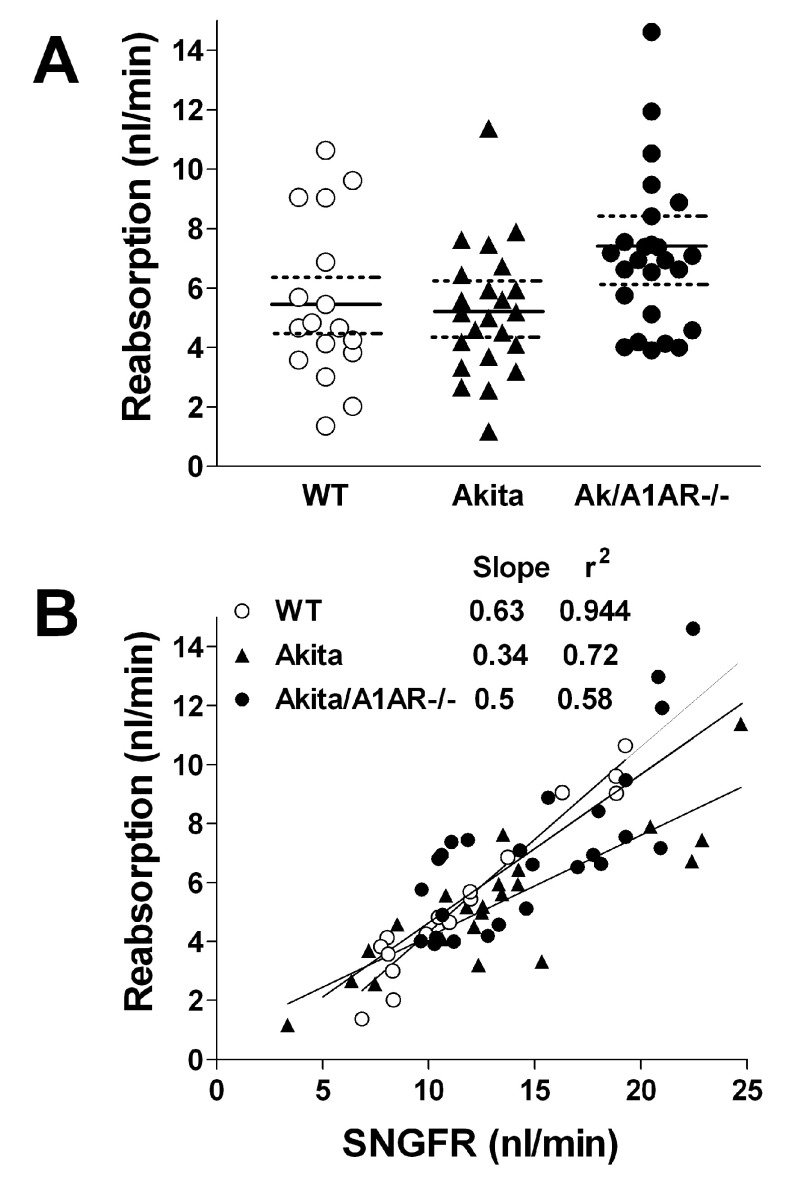
Measurements of proximal fluid reabsorption in wild type (WT), Akita, and Akita/A1AR-/- double mutant mice (Ak/A1AR-/-). **A**: Fluid absorption rates in individual nephrons; solid lines are means, and broken lines are 95% confidence intervals. **B**: Relationship between SNGFR and reabsorption rate; lines indicate linear regressions.

Measurements of SNGFR and fluid reabsorption along the proximal tubule by micropuncture confirmed the presence of hyperfiltration at the single nephron level ( Figure 1A). Mean SNGFR was 11.8 ± 1 nl/min in WT (n=17), 13.05 ± 1.1 nl/min in Akita (n=23; p=0.27), and 15.4 ± 0.84 nl/min in Akita/A1AR-/- mice (n=26; p=0.009 compared to WT; p=0.09 compared to Akita). The 10.6% and 23% rise of SNGFR in Akita and double mutant mice respectively was less than the 24.8% and 56% increments of whole kidney GFR. Fractional fluid absorptions expressed as the ratio of iothalamate concentration in tubular fluid (TF) over that in plasma (P) (TF/Piothalamate) ( Figure 1B) or converted to fractional fluid reabsorption in percent of GFR were not significantly different between WT and diabetic animals, averaging 1.84 ± 0.07 or 44.3 ± 2.3% in WT, 1.72 ± 0.05 or 40.7 ± 1.8% in Akita (p=0.27) and 2.1 ± 0.1 or 49.6 ± 2.3% in Akita/A1AR double mutant mice (p=0.06 compared to WT). Fluid reabsorption in absolute terms ( Figure 2A) was not significantly different between WT and diabetic mice, but a tendency for slightly higher SNGFR and TF/Piothalamate values in the Akita/A1AR-/- mice added up to a significantly higher reabsorption rate compared to Akita mice (p<0.05 by ANOVA). Glomerulotubular balance, the relationship between GFR and reabsorption, was not disrupted and was not markedly different in the three strains of mice ( Figure 2B).

Single experiments in Akita mice Units length of fluid column, nl total fluid volume collected, min collection time, flow flow rate (nl/min), Ucpm I125 counts per minute in total fluid volume, Bkg I125 background, column H net cpm (Ucpm-Bkg), TF tubular fluid (cpm/nl). P plasma (cpm/nl), TF/P ratio tubular fluid cpm divided by plasma cpm, SNGFR single nephron GFR (TF/P x flow rate), absorption fluid reabsorption (SNGFR - flow rate), % Del fluid delivered to collection site in % of SNGFR, % Abs fluid absorption in % of SNGFR, MAP mean arterial pressure during collection. U urine (cpm in 2 duplicates), P plasma (cpm in 2 samples in duplicates).

Single experiments in Akita/A1AR-/- (DKO) mice Units length of fluid column, nl total fluid volume collected, min collection time, flow flow rate (nl/min), Ucpm I125 counts per minute in total fluid volume, Bkg I125 background, column H net cpm (Ucpm-Bkg), TF tubular fluid (cpm/nl). P plasma (cpm/nl), TF/P ratio tubular fluid cpm divided by plasma cpm, SNGFR single nephron GFR (TF/P x flow rate), absorption fluid reabsorption (SNGFR - flow rate), % Del fluid delivered to collection site in % of SNGFR, % Abs fluid absorption in % of SNGFR, MAP mean arterial pressure during collection. U urine (cpm in 2 duplicates), P plasma (cpm in 2 samples in duplicates).

Single experiments in wild type (WT) mice Units length of fluid column, nl total fluid volume collected, min collection time, flow flow rate (nl/min), Ucpm I125 counts per minute in total fluid volume, Bkg I125 background, column H net cpm (Ucpm-Bkg), TF tubular fluid (cpm/nl). P plasma (cpm/nl), TF/P ratio tubular fluid cpm divided by plasma cpm, SNGFR single nephron GFR (TF/P x flow rate), absorption fluid reabsorption (SNGFR - flow rate), % Del fluid delivered to collection site in % of SNGFR, % Abs fluid absorption in % of SNGFR, MAP mean arterial pressure during collection. U urine (cpm in 2 duplicates), P plasma (cpm in 2 samples in duplicates).

Data Summary of measurements of renal function in kidneys and single nephrons of wild type, Akita, and Akita/A1AR-/- mice Units length of fluid column, nl total fluid volume collected, min collection time, flow flow rate (nl/min), Ucpm I125 counts per minute in total fluid volume, Bkg I125 background, column H net cpm (Ucpm-Bkg), TF tubular fluid (cpm/nl). P plasma (cpm/nl), TF/P ratio tubular fluid cpm divided by plasma cpm, SNGFR single nephron GFR (TF/P x flow rate), absorption fluid reabsorption (SNGFR - flow rate), % Del fluid delivered to collection site in % of SNGFR, % Abs fluid absorption in % of SNGFR, MAP mean arterial pressure during collection. U urine (cpm in 2 duplicates), P plasma (cpm in 2 samples in duplicates).

## Discussion

**Figure 3.**
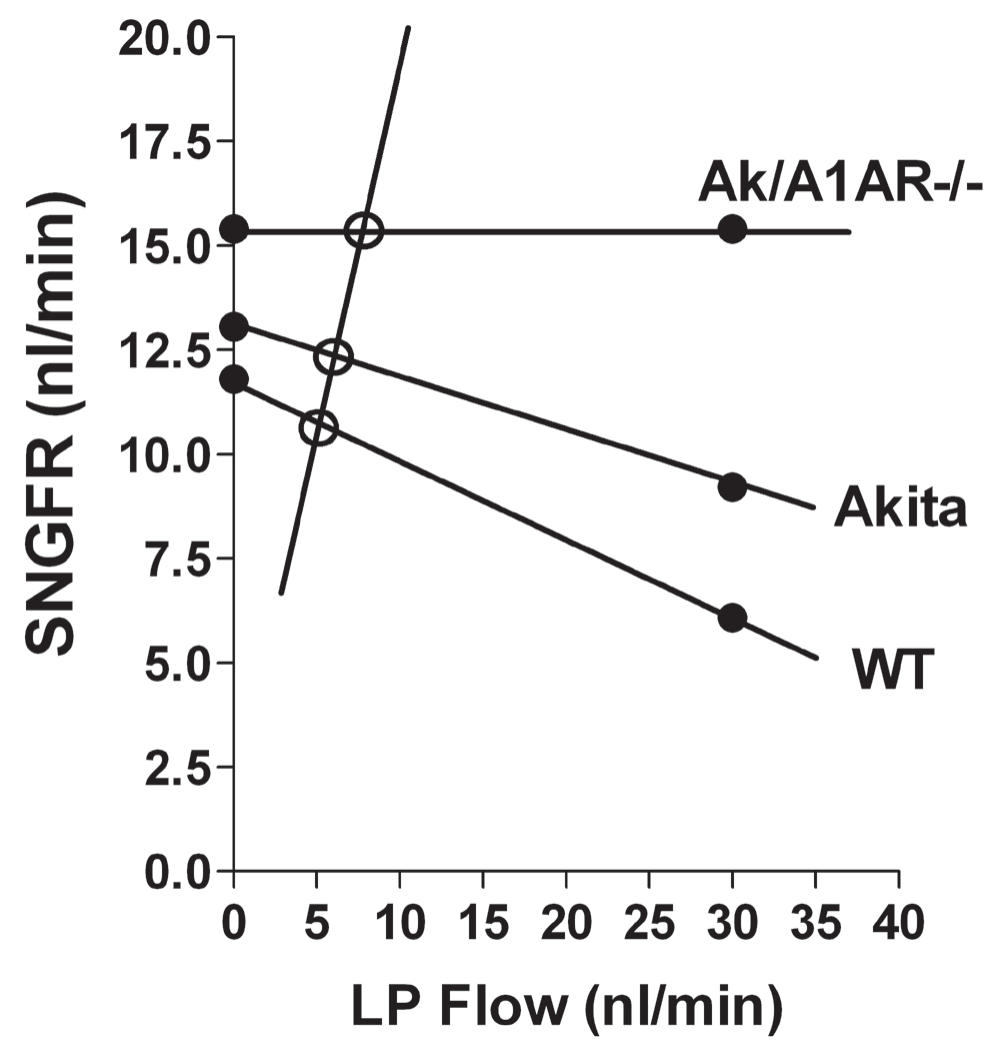
Relationship between the flow rate at the end of the proximal tubule (LP Flow) and SNGFR in wild type (WT), Akita, and Akita/A1AR-/- mice. SNGFR values (black dots) on the y axis represent SNGFR values from the present experiments; SNGFR values at LP flow of 30 nl/min come from our earlier study in which the effect of raising LP flow on early proximal flow rate was determined ^9^. The negative lines connecting SNGFRs represent the TGF relationship that, for reasons of simplicity, is drawn as a linear relation, although it is known to be sigmoidal ^19, 20^. The slope of the positive line reflects proximal reabsorption, and it was calculated assuming a TF/Piothalamate ratio of 2. The intersects between the TGF and reabsorption relationships indicated by the open circles represent steady-state values for reabsorption and SNGFR ^19^.

Previous measurements of GFR in conscious young animals have shown that type I diabetic Akita mice tend to show hyperfiltration that became highly significant in the A1AR-null genetic background ^12^. Similarly, the induction of diabetes by alloxan was associated with hyperfiltration in both WT and A1AR-/- mice ^13^. The present results confirm these observations in anesthetized animals in which the GFR of Akita mice increased by 25% in the C57Bl/6 background (nonsignificant) and by 56% (p<0.05) in the A1AR-/- background. As we have argued previously, the augmented hyperfiltration cannot be mediated by TGF since the A1AR-deficiency in both mixed WT and Akita diabetic genetic backgrounds renders TGF non-functional ^9, 12, 14^. We cannot exclude the possibility that A1AR-deficiency directly enhanced GFR through some unknown mechanism. However, A1AR-deficient mice have been shown previously to have normal filtration rates ^9, 15^ so that the GFR-raising effect would have to be linked to A1AR deficiency under diabetic conditions. The present micropuncture results corroborate the irrelevance of TGF for hyperfiltration in diabetic Akita mice in another way. Hyperfiltration at the single nephron level was seen during withdrawal of fluid at late proximal tubular sites, thereby preventing fluid from gaining access to sites beyond the proximal tubule and effectively normalizing distal fluid delivery to zero. Thus, by eliminating TGF influences, this opening of the feedback loop reveals TGF-independent effects on GFR. To the extent that TGF is functional in a given animal or condition, SNGFR measured by proximal fluid collections represents a non-steady-state that overestimates SNGFR by acute removal of the GFR-suppressing action of TGF ( Figure 3). In the present experiments, one may assume that SNGFR is closest to steady-state values in the Akita/A1AR-/- mice in which TGF is non-functional under all circumstances, and that proximal SNGFRs in WT and Akita mice overestimate true filtration rates to probably different degrees. We suggest that this overestimation is the reason why the relative changes of SNGFR in diabetic mice compared to WT are less than those of kidney GFR (10.6% and 23% vs. 25% and 56% in Akita and Akita/A1AR-/-, respectively). Our data are consistent with the notion that the rise of SNGFR in Akita/A1AR-/- mice is primary, and that it is made possible by absence of a TGF-mediated vasoconstrictor input. The tendency for GFR and SNGFR to increase in the Akita mice on a C57Bl/6 background may reflect the reduced TGF efficiency previously observed in both type I and type II diabetes ^9, 16, 17^. Our study was not designed to identify the factors responsible for the increased filtration rate. Nevertheless, it is noteworthy that kidney GFR was not significantly different between control and diabetic animals when GFR was related to kidney weight. As also documented in the present studies, renal hypertrophy out of proportion to body weight is a well known early symptom of diabetes mellitus in patients, and this growth includes an increase in glomerular capillary surface area and thus presumably in the filtration coefficient ^18^.

The issue of whether proximal fluid reabsorption is overwhelmed in Akita mice cannot be decided authoritatively due to the non-steady-state conditions and limited statistical power. Nevertheless, the present experiments did not show a significant reduction in either fractional or absolute proximal fluid reabsorption in Akita mice compared to WT mice (40.7 vs. 44.3% and 5.4 vs. 5.2 nl/min). The relative increase in plasma glucose levels of Akita mice at a young age is about threefold based on previous measurements ^9^, from about 200 to 600 mg/dl, and therefore comparable to what has been reported in the streptozotocin model of diabetes in rats, except that baseline glucose in C57Bl/6 mice was higher ^7, 17^. The increase of proximal absorption in Akita/A1AR double mutant mice is for the most part due to the increase of SNGFR, reflecting the maintenance of load-dependent fluid reabsorption ( Figure 2B). The inability to detect clear GFR-independent changes in proximal fluid reabsorption in our study is consistent with previous evidence that wide variations of glucose reabsorption rates have little effect on net fluid retrieval. For example, severe reductions of proximal glucose transport in SGLT2-deficient mice were not associated with significantly reduced proximal fluid fluxes ^21, 22^. Conversely, raising plasma glucose by infusion has little effect on proximal fluid reabsorption consistent with mathematical modeling, showing small and biphasic effects of glucose on water flux over a three- to fourfold variation of glucose around normal values ^23, 24^. While the links between Na and glucose uptake and between Na and water transport demand that fluid and glucose absorption rates should co-vary, the magnitude of the estimated effects is too small to be detectable by micropuncture.

In summary, our results confirm at the single nephron level that GFR increases in diabetic Akita mice as a function of TGF non-functionality, consistent with the notion that TGF prevents hyperfiltration in this model of type I diabetes. Rates of absolute and fractional fluid reabsorption were found to be comparable between control and diabetic animals. While hyperfiltration is seen in both streptozotocin-induced diabetes and in the genetic diabetes of the Akita mice, the mechanisms underlying this functional abnormality may be different in the two models.
