# Supplementary material for: Nephron filtration rate and proximal tubular fluid reabsorption in the Akita mouse model of type I diabetes mellitus
Source: F1000Res. 2013 Mar 11;2:83. [Version 1] doi: 10.12688/f1000research.2-83.v1 (PMC3814914; doi:10.12688/f1000research.2-83.v1)
Supplement: Peer review history

# Peer review: Referee response for version 1

**Published:** 2013-03-18

**Authors:** Bellamkonda Kishore

**Affiliations:** 1 Department of Internal Medicine (Nephrology), VA Medical Center & University of Utah, Salt Lake City, UT, USA

The manuscript by Schnermann and associates entitled, “Nephron filtration rate and proximal tubular fluid reabsorption in the Akita mouse model of type I diabetes mellitus” addresses a critical question in the development of diabetic nephropathy. To address the critical question of the role of TGF in the glomerular hyperfiltration in diabetes nephropathy, the authors used transgenic mouse models.  Diabetic nephropathy in human patients is a complex disease and no single transgenic mouse model of diabetic nephropathy can perfectly match the human disease. Despite these limitations, Schnermann and associates defined well the problem they addressed, and used appropriate mouse models of type I diabetes mellitus and skilled techniques. In this context, their approach is well controlled and provided meaningful data vis-à-vis the problem they attempted to address. Thus, overall it is convincing to this reviewer that their conclusions are valid and acceptable to the scientific community. I do not have any major issues, and only present a few minor concerns.

- Although, the manuscript is well written, at some critical places the use of long sentences is not advisable to convey the meaning smoothly, especially to the not-so-experienced readers and junior scientists.

- It is not clear to me whether +/- data presented in the Table 1 represent SD or SEM. It should be mentioned in the Table footnote as well as under the Statistics description.

- Although the scattered data points show the trends, however, looking at the Akita group in Fig 1A, and Ak/A1AR-/- group in Fig 1B and 2A, it is clear that the distribution is not normal.  The Ak/A1AR-/- group has distinctive distributions as compared to the other two groups in these figure panels.  Hence, the data presented in Figures 1 & 2 will be better interpretable by including parallel box plots. The box plots also provide more insights by showing the median and quartiles.  The authors should consider this suggestion.

I have read this submission. I believe that I have an appropriate level of expertise to confirm that it is of an acceptable scientific standard.

# Response

**Published:** 2013-04-02

**Authors:** Jurgen Schnermann

**Affiliations:** Kidney Disease Section, National Institute of Diabetes and Digestive and Kidney Diseases, USA

Dear Dr. Kishore,

Thanks for your comments!

You talk about model limitations in your introduction, and I wanted to confirm that we agree and are fully aware of this. One of the most intriguing aspects of the human disease is its variability in that some diabetics have hyperfiltration and some don’t, and some of those that do may get renal disease and some don’t. I am not aware that any of the experimental models mimic this critical phenotype, in part perhaps because the statistical database isn't large enough to show it. In any case, the mechanism of hyperfiltration, something that one may get out of animal studies, is much less important than its predictive value for developing renal disease, and this one can get only out of human studies. Thus, hyperfiltration in mouse models (and the mechanisms causing it) may be more of academic than clinical interest. Certainly the question of whether an increase of plasma glucose into the diabetic range increases or decreases proximal tubular fluid reabsorption is a defined question that should be answerable, and the impression that even this limited question appears to be model-dependent is somewhat disappointing.The deviations presented in Table 1 are SEMs. In regard to the figures, we thought that by showing every single measurement we leave the issue of data distribution open. Box plots don’t add much other than 25% and 75% quartiles and minimum and maximal values, and I simply doubt that there is much information in this. As to distribution, we agree that some of the data look not normally distributed, but to conclude that this is an inherent property of one strain vs. another is most likely wrong. It is much more likely that data from the 7th, or 8th, or 9th or nth mouse would fill the void and make the data more and more normally distributed. 

In regard to the long sentences, we know we are no Shakespeares, but the writing does not seem too bad – comparatively.

# Peer review: Referee response for version 1

**Published:** 2013-03-13

**Authors:** Helle Praetorius

**Affiliations:** 1 Aarhus University, Aarhus, Denmark

The manuscript 'Nephron filtration rate and proximal tubular fluid reabsorption in Akita mouse model of type I diabetes mellitus' is a follow up to the same groups previous publication on hyperfiltration in diabetic mice model. The manuscript is very clear and easy to read despite the relatively complicated content. It addresses essential points for the nature of DM- induced hyperfiltration and the conclusion of TGF as a relative protector against DM-induced hyperfiltation is well founded. In this paper the authors look at younger mice, which is commendable as the hyperfiltration is seen as an early event in diabetes that precedes significant DM-induced renal failure. The experiments are of a high quality and the figures are generally well prepared.

Minor concerns:

- The current manuscript builds on previous results that firmly substantiate the used diabetic models. In this study the age of the mice are not completely comparable to the previous study. It would, however, be helpful if it was clear from the text that the relevant data is already available - that the animals in fact have high blood glucose and are seemingly slightly dehydrated.
   
- It is unclear where the data comes from in Figure 3 (30 nl/min). The legend states that the values come from reference 9 - but the mean values for EPFR given in that publication is: (5.0 nl/min - WT; 6.9 nl/min - Ins ^+/-^; 11.5 nl/min Ins ^+/-^A1AR ^-/-^) – so this must apparently be new data for the 30 nl/min? – or is there some conversion of the numbers that is not perfectly clear. This point is relatively important as a fall from 15.2 nl/min (current value, 0 nl/min) to 11.5 nl/min (value from old paper, 30 nl/min) in the Ak/A1AR ^-/- ^is quite substantial and does witness about some TGF in this mouse. 
   
- Moreover, what is the age of the animals (30 nl/min) that are compared here?  Is it reasonable to make a line between the two points if they are indeed measured in separate experiments.
- It is not explicit that the **n** in the measurements of SNGFR is number of experiments and not animals.
   
- The test used to test for normal distribution of the data, which is a prerequisite for using the ANOVA test, is not stated under statistics.
   
- The values in the text are not given with the same number of decimals.
   
- The method for measuring GFR in general is not included in the method section.

I have read this submission. I believe that I have an appropriate level of expertise to confirm that it is of an acceptable scientific standard.

# Response

**Published:** 2013-03-14

**Authors:** Jurgen Schnermann

**Affiliations:** Kidney Disease Section, National Institute of Diabetes and Digestive and Kidney Diseases, USA

Dear Dr. Praetorius, 

     

We appreciate your comments, and provide the following in response to the concerns that you have raised:   

The youngest cohort of mice in our previous study ( Faulhaber-Walter *et al.*) had an age of 14 weeks. While the age-matching between strains in the present experiments was not perfect, the mean of the ages is about 14 weeks. According to our previous data diabetic mice at this age have plasma glucose levels of between 600 and 700 mg/dl. This is an agreement with the original description of the Akita mice, in which diabetes had been shown to be of early onset with an approximate threefold increase of plasma glucose by 7 weeks of age ( Yoshioka *et al.*). A manifestation of diabetes in the current experiments is the increased urine flow, which is a consequence of non-absorbed glucose as qualitatively established by dipstick, which affirms that hyperglycemia and the increase of filtered glucose must have exceeded the maximal glucose absorptive capacity.

 

Figure 3 was meant to facilitate the understanding of the non-steady-state problem in SNGFR measurements in the proximal tubule rather than being a presentation of hard data. Nevertheless, the endpoint values at 30 nl/min were derived from reductions of the current SNGFR values by the relative EPFR decrements seen in the different strains previously which were 48% in wild type, 29% in Akita, and 0% in the double mutants. One might also point out that even if TGF didn’t exist at all and the SNGFRs were in fact steady-state values, the main argument could still be made in that measured GFR’s increased in the diabetic mice (at least in those without A1AR). The ages of the animals used at that time were between 9.8 and 17 weeks.                

It is hard to affirm Gaussian distribution if the sample size is relatively small, but all groups passed the KS test for normality. Nevertheless, subjecting the SNGFR data to a nonparametric test (Kruskal-Wallis) showed that the probability of the null hypothesis to be correct, 0.026 suggesting that the medians between the groups are in all likelihood different.
